# Expansion of the distribution range of *Aspleniumtrilobum* Cav (Polypodiopsida, Aspleniaceae) in the Mediterranean forest of the Chilean coast

**DOI:** 10.3897/BDJ.11.e105990

**Published:** 2023-07-17

**Authors:** Jimmy Pincheira-Ulbrich, Ulises Zambrano, Felipe Contreras

**Affiliations:** 1 Nucleo de Estudios Ambientales, Universidad Católica de Temuco, Temuco, Chile Nucleo de Estudios Ambientales, Universidad Católica de Temuco Temuco Chile; 2 Laboratorio de Planificación Territorial, Universidad Católica de Temuco, Temuco, Chile Laboratorio de Planificación Territorial, Universidad Católica de Temuco Temuco Chile; 3 Departamento de Ciencias Ambientales, Facultad de Recursoso Naturales, Universidad Católica de Temuco. Rudecindo Ortega 02950, Temuco, Chile Departamento de Ciencias Ambientales, Facultad de Recursoso Naturales, Universidad Católica de Temuco. Rudecindo Ortega 02950 Temuco Chile; 4 Universidad Católica de Temuco, Facultad de Recursos Naturales, Geografía, Temuco, Chile Universidad Católica de Temuco, Facultad de Recursos Naturales, Geografía Temuco Chile

**Keywords:** biodiversity hostpost, epiphyte, queule, sclerophyllous forest, wetland

## Abstract

The biodiversity hotspot of central Chile is home to a high proportion of endemic species, but some of these species are inconspicuous and not easily observed. During a botanical exploration in the Los Queules National Reserve (Chile), a population of *Aspleniumtrilobum* Cav. was identified. The plants were found growing on the bark of a *Myrceugeniaparvifolia* (DC.) Kausel tree in a small swamp next to specimens of *Drimyswinteri* J.R.Forst. & G.Forst. (35°59'11.84"S; 72°41'11.53"W). Several previously unrecorded species were found, including Carexcf.excelsa Poepp. ex Kunth, *Chusquea* cf. *quila Kunth*, Ercillacf.spicata (Bertero) Moq., and *Boquilatrifoliolata* (DC.) Decne., highlighting the importance of exploring and documenting this biodiversity hotspot. The discovery in this wilderness area extends the distribution 86 km north on the continent, which was previously limited to the east of the municipality of Penco in the Biobío region (36°44'9.26"S; 72°57'42.5"W). This paper presents an observed specimen, its locality, and associated species.

## Introduction

With more than 750 species worldwide, *Asplenium* L. is one of the fern genera with the greatest species diversity and geographical distribution. This genus and *Hymenasplenium* (at least 66 species) comprise the family Aspleniaceae, with Asplenium known for its remarkable species richness (PPGI 2016, Hassler 2023).

In Chile, eleven species belonging to the genus Asplenium have been documented (Rodríguez et al. 2009). Among them, *Aspleniumtrilobum* Cav. is a fern species endemic to the sub-Antarctic rainforests of Chile and Argentina (Rodríguez et al. 2009, Ganen et al. 2017). *A.trilobum* is found at altitudes ranging from 5 to 600 m a.s.l., predominantly as an epiphyte that primarily inhabits tree trunks in shaded areas with high humidity.

The known distribution of this species has its northern limit in the Municipality of Penco, on the coast of the Biobío Region (36°44'9.26"S; 72°57'42.5"W), while the southern limit is in Laguna San Rafael, Aysén Region (46°40'S; 73°50'W) (pers. comm. Alicia Marticorena, curator of the CONC herbarium, see also Rodríguez 1995). In central Chile, A.trilobum has been classified as a threatened species by the Chilean Ministry of the Environment, highlighting the urgency of its conservation (MMA 2022).

*Aspleniumtrilobum* is distinguished by its 2 to 6 cm long rhombic laminae, often with an irregular lobe, contrastingly coloured with deep green on the upper surface and a paler, almost glaucous green beneath. Supported by a stipe of equal or sometimes greater length, the laminae's cuneate and entire base transition to small rounded or serrate teeth. Flabellate venation features 2 to 6 sori, each 0.5 to 1 cm long, flanking the rachis, with a persistent lateral indusium. The frond is completed by a slender, glabrous petiole, devoid of pilosity (Looser 1944, Rodríguez and Baeza 1991, Rodríguez 1995, Ganen et al. 2017).

The recent discovery of *A.trilobum* in the Los Queules National Reserve, located in the Mediterranean forest of central Chile, a global biodiversity hotspot, highlights the need for biodiversity monitoring and inventories, an unfulfilled need in Latin America (Myers et al. 2000, Mittermeier et al. 2011). Species inventories form the basis for improving our understanding of the distribution and ecology of species present in the region, and facilitate the implementation of appropriate conservation and management strategies to protect these ecosystems and their biodiversity.

This finding is particularly relevant in the context of global climate change and landscape fragmentation, as the central Chilean coast has been drastically altered by the almost complete replacement of native vegetation by forestry plantations and agricultural crops. These human activities have led to significant habitat loss and a decline in the region's biodiversity (Bustamante and Castor 1998, Echeverria et al. 2006).

In this paper, an observed specimen of *A.trilobum* is presented, the locality and associated species are described, and the significance of this discovery for the coastal distribution of the Mediterranean forest is highlighted.

## Study site: Los Queules National Reserve

**Figure 1.**
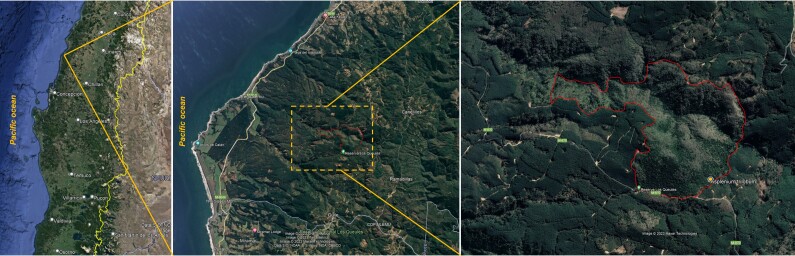
Los Queules National Reserve (satellite image from Google Earth).

The Los Queules National Reserve is administratively located in the municipality of Pelluhue, in the Maule region (Fig. 1). It covers an area of 147 hectares and is located in a matrix of coastal forest plantations. The name "Keule" refers to an endemic and threatened species in Chile (*Gomortegakeule* (Molina) Baill.). The forest on the hilltops is mainly dominated by *Nothofagusglauca* (Phil.) Krasser and *Nothofagusobliqua* (Mirb.) Oerst. In the wetter areas, species such as *Aextoxiconpunctatum* Ruiz & Pav., *Cryptocaryaalba* (Molina) Looser and *Persealingue* (Ruiz & Pav.) Nees make up the dominant canopy layer.

## The new record

During a botanical exploration carried out on 4 October 2022 in the Los Queules National Reserve (Maule Region, Chile; Fig. 1), 19 fronds, presumably at least partly connected by rhizomes and thus corresponding to an unknown number of individual plants fronds of *A.trilobum* (Fig. 3), were observed in a small swampy wetland (35°59'11.84"S; 72°41'11.53"W). The fronds were found on the northwestern face of the trunk of a *Myrceugeniaparvifolia* (DC.) Kausel individual with a remarkably large diameter of about 40 cm at breast height, which is an unusual attribute for this species, located at 404 m a.s.l. (Fig. 2). The discovery in this wilderness area extends the distribution in Chile by 86 km to the north, which was previously limited to the east of the municipality of Penco (36°44'9.26''S / 72°57'42.5''W) (e.g. Rodriguez et al. 2018, Rodríguez 1995). The specimen has been deposited in the CONC herbarium under code 192770.

The site is located on the eastern edge of the wilderness area, about 60 metres from forest plantations. The accompanying species were *Parablechnumchilense* (Kaulf.) Gasper & Salino, *Luzuriagapolyphylla* (Hook.) J.F. Macbr., Carexcf.excelsa Poepp. ex Kunth, Chusqueacf.quila Kunth, *Myrceugeniaexsucca* (DC.) O. Berg, *Myrceugeniaparvifolia* (DC.) Kausel, *Drimyswinteri* J.R. Forst. & G. Forst., *Hydrangeaserratifolia* (Hook. & Arn.) F. Phil., *Ercillacf.spicata* (Bertero) Moq., *Rhamnusdiffusus* Clos, *Boquilatrifoliolata* (DC.) Decne., and *Ugnicandollei* (Barnéoud) O. Berg.

It is important to note that Carexcf.excelsa (Fig. 2a), Chusqueacf.quila (Fig. 2a), Ercillacf.spicata (Fig. 5) and *Boquilatrifoliolata* (*Fig. 4*) are new records for this wilderness area (Arroyo et al. 2005). The species identified as "cf." in this study indicate that the identification of these species is tentative, as reproductive structures were not observed at the time of sampling, making accurate identification difficult. The presence of *Ercillaspicata* in the study area is highly plausible, as it is one of only two species within its genus. *Chusqueaquila* is also highly possible, as its morphology is clearly different from the only Chusquea species previously recorded at the site: *C.cumingii*. However, the identification of these species still needs to be confirmed. In a similar case, although *Carexaphylla* Kunth and *Carexexcelsa* L. have been reported from nearby wild areas (Arroyo et al. 2005), the plant found in this swamp habitat seems more consistent with *C.excelsa*. If confirmed, this would be the first record of this genus at the site.

## Importance of botanical explorations

The importance of botanical exploration in advancing our taxonomic knowledge is inestimable. However, many biodiversity studies have shown a marked accessibility bias, with sampling favouring areas close to major roads and other access routes, leaving interior and remote regions largely undersampled (Bebber et al. 2010, Hortal et al. 2015, ter Steege et al. 2016). This sampling bias may result in the omission of rare or restricted species (Gotelli and Colwell 2001).

New species and distribution records are frequently detected in previously explored areas (e.g. Joppa et al. 2010, Pincheira-Ulbrich et al. 2022, Villarroel et al. 2022). This highlights the need to intensify sampling efforts, both in remote areas and in accessible regions that have also been under-sampled (e.g., Meyer et al. 2016, Pincheira-Ulbrich et al. 2021). Plant taxonomy, although fundamental to understanding biodiversity, faces serious challenges due to the scarcity of experts (Hopkins and Freckleton 2002, Wheeler et al. 2004, Rouhan and Gaudeul 2020).

In the context of this study, it is possible to suggest that the distribution of *A.trilobum* in Chile may be more extensive than we know. The discovery of *A.trilobum* in the Los Queules National Reserve, and the detection of other previously unrecorded species in the area, underlines the existence of a diversity and distribution of species yet to be explored and understood in these ecosystems. Consequently, the development of inventories and basic research in under-explored areas, such as ravines and forest remnants, should be promoted. Finally, it is crucial to encourage the training of advanced human capital in botany, taxonomy and genetics, areas that have received little attention from the Chilean State.

## Figures and Tables

**Figure 2a.**
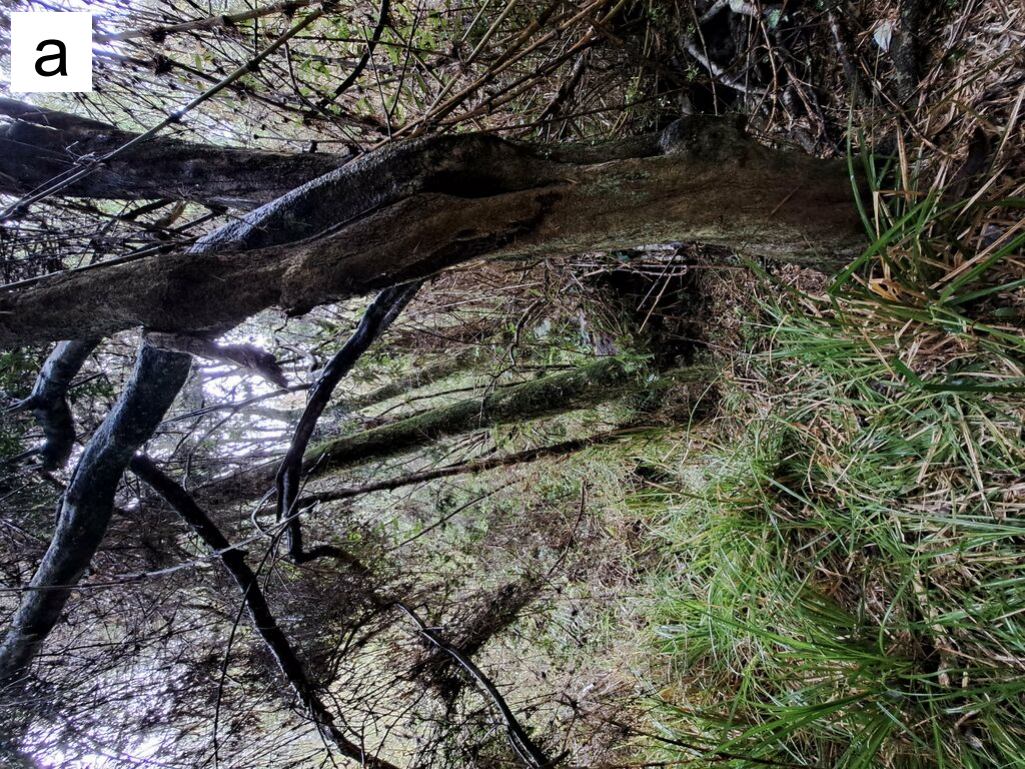
In the centre of the picture, there is a specimen of *Myrceugeniaparvifolia* and associated species including Chusqueacf.quila and Carexcf.excelsa

**Figure 2b.**
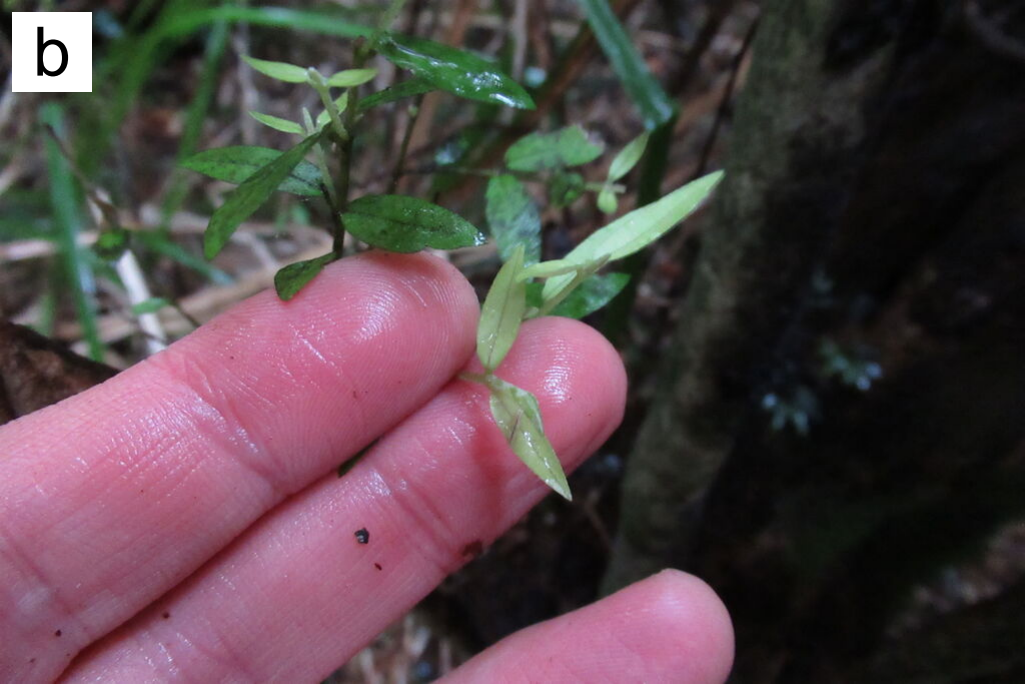
Detail of *M.parvifolia* leaves

**Figure 3a.**
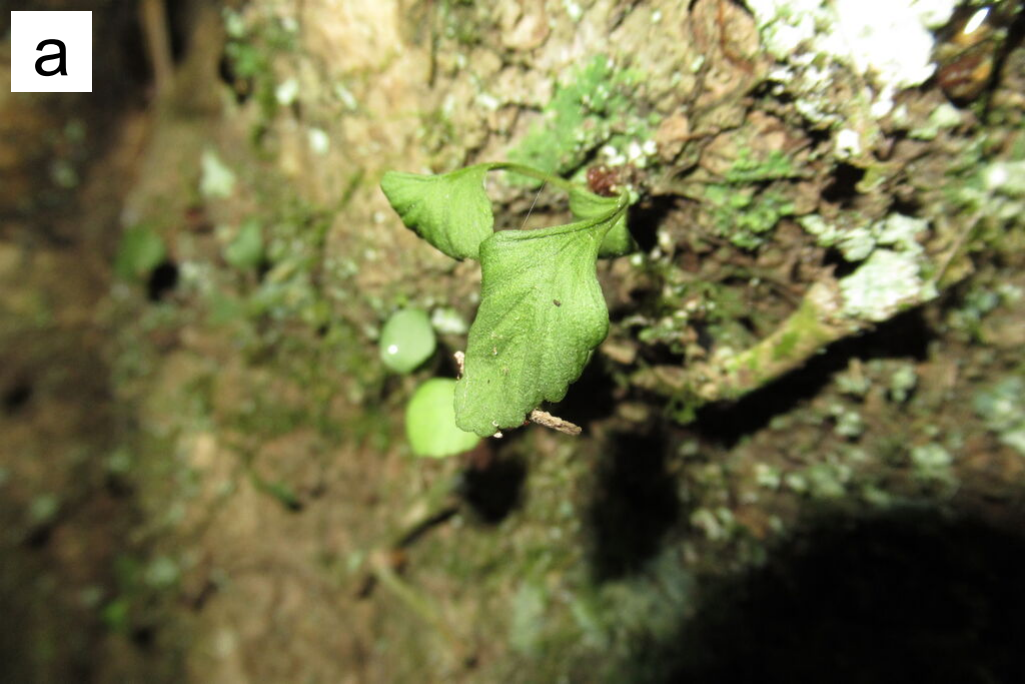
Frond adaxial surface (phototography taken with flash)

**Figure 3b.**
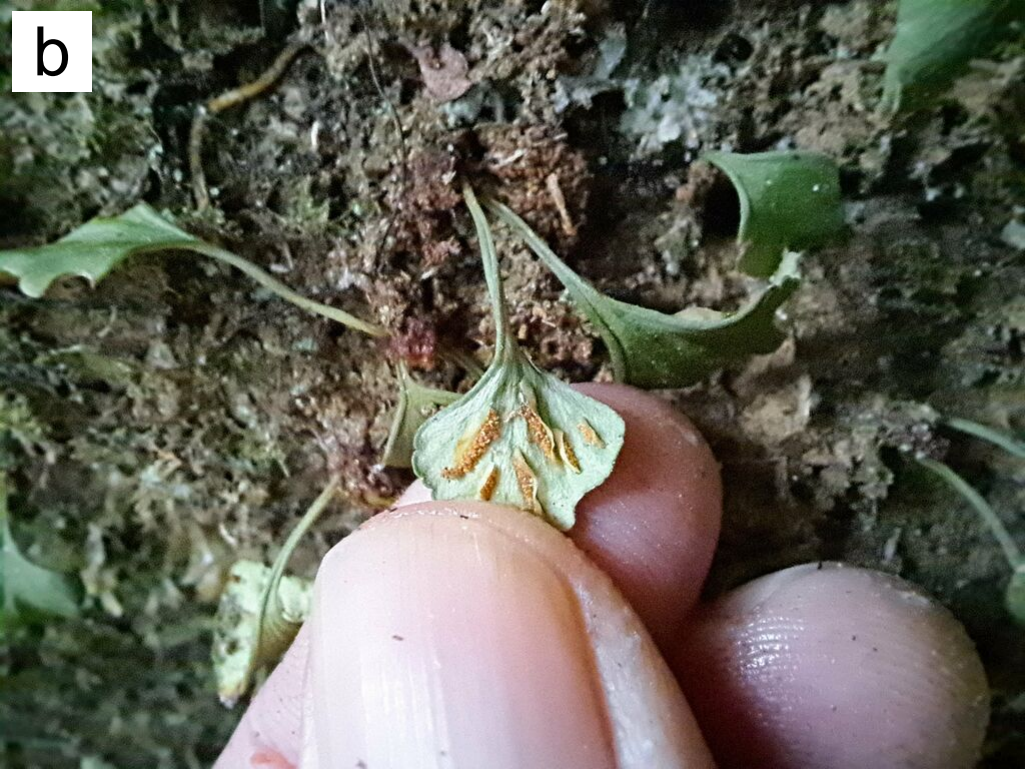
Sori on the abaxial surface

**Figure 3c.**
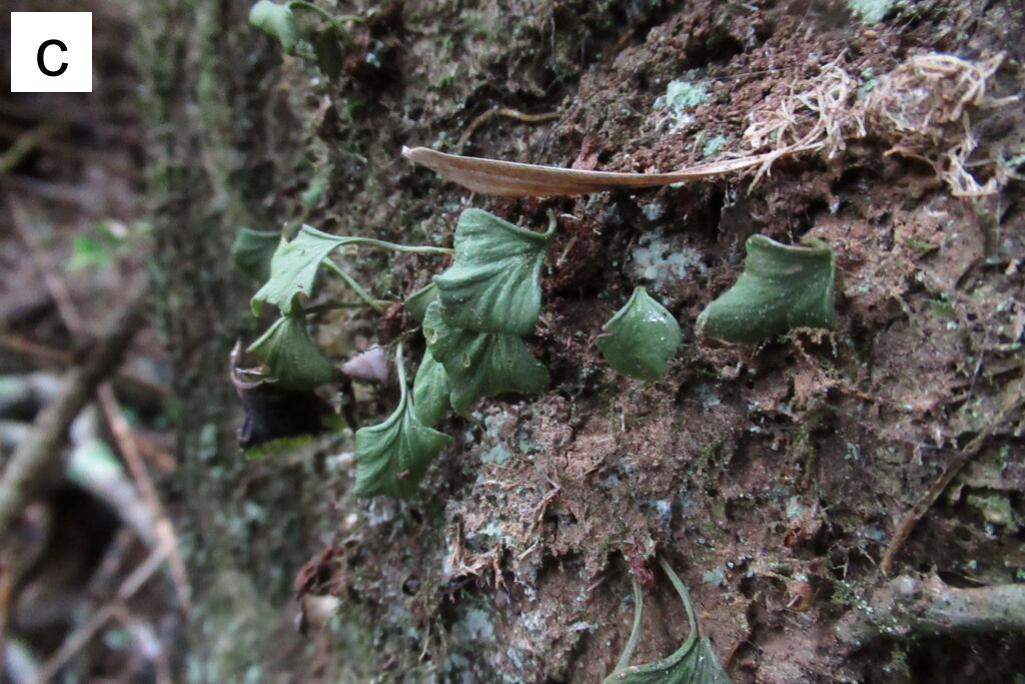
Fronds taken in natural light

**Figure 3d.**
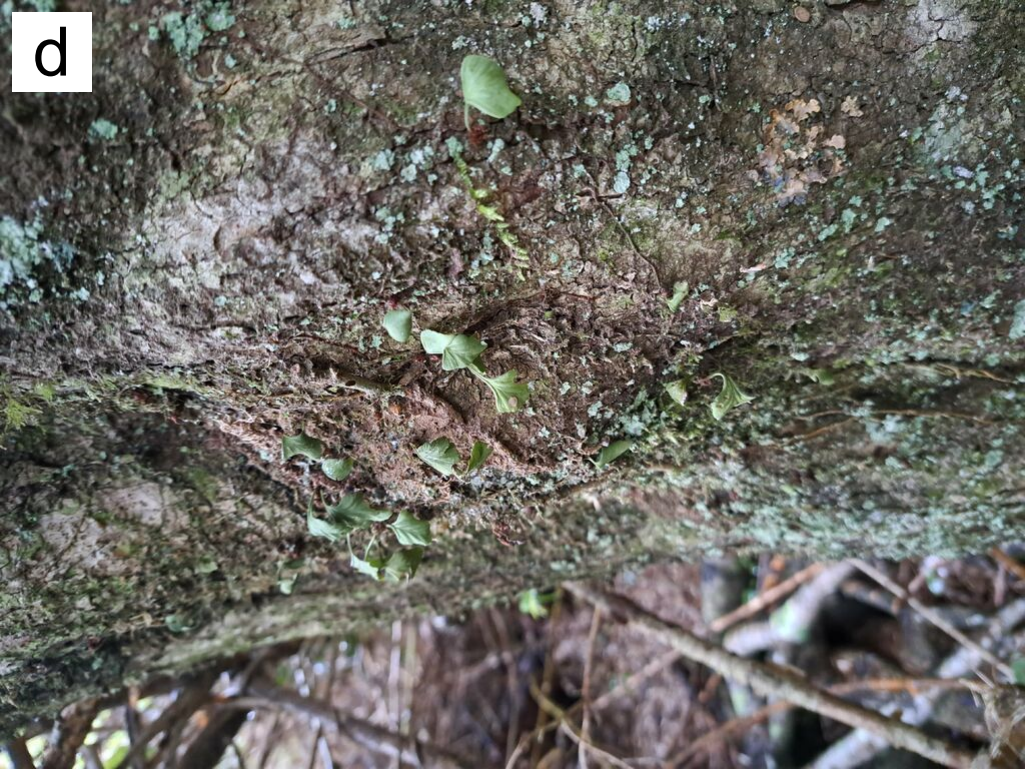
Fronds photographed from a perspective that allows their position on the trunk to be appreciated.

**Figure 4a.**
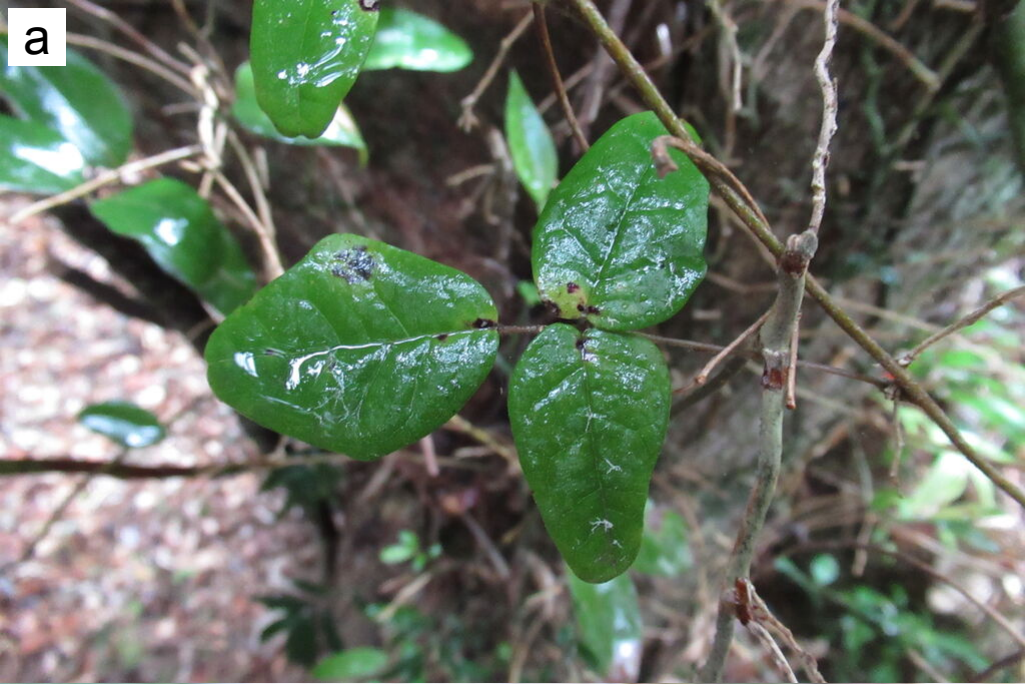
Leaves

**Figure 4b.**
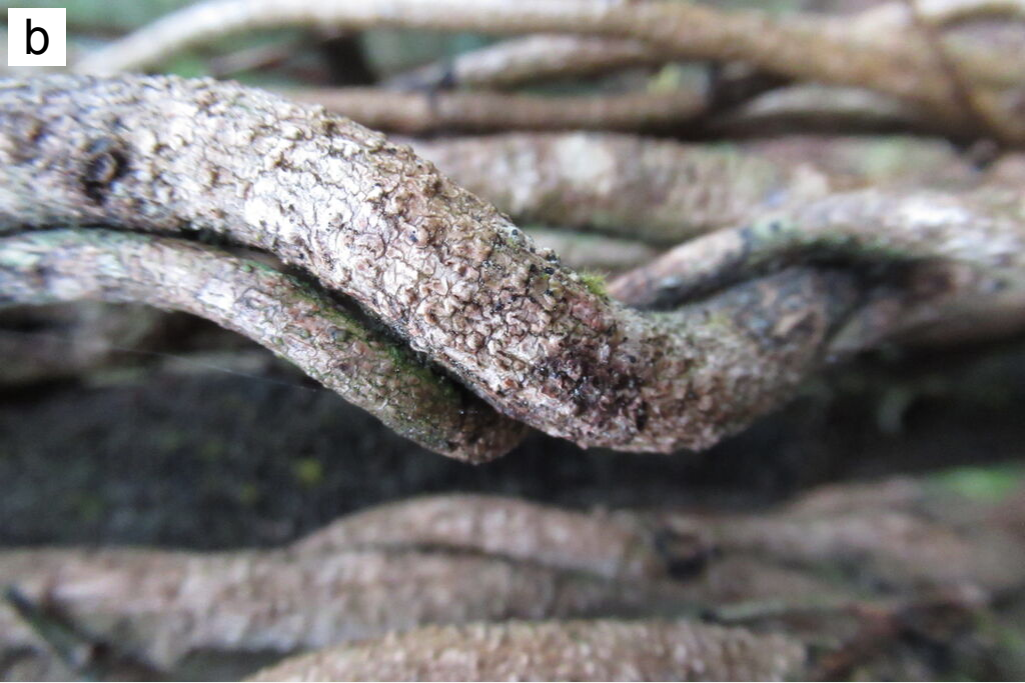
Stem

**Figure 5a.**
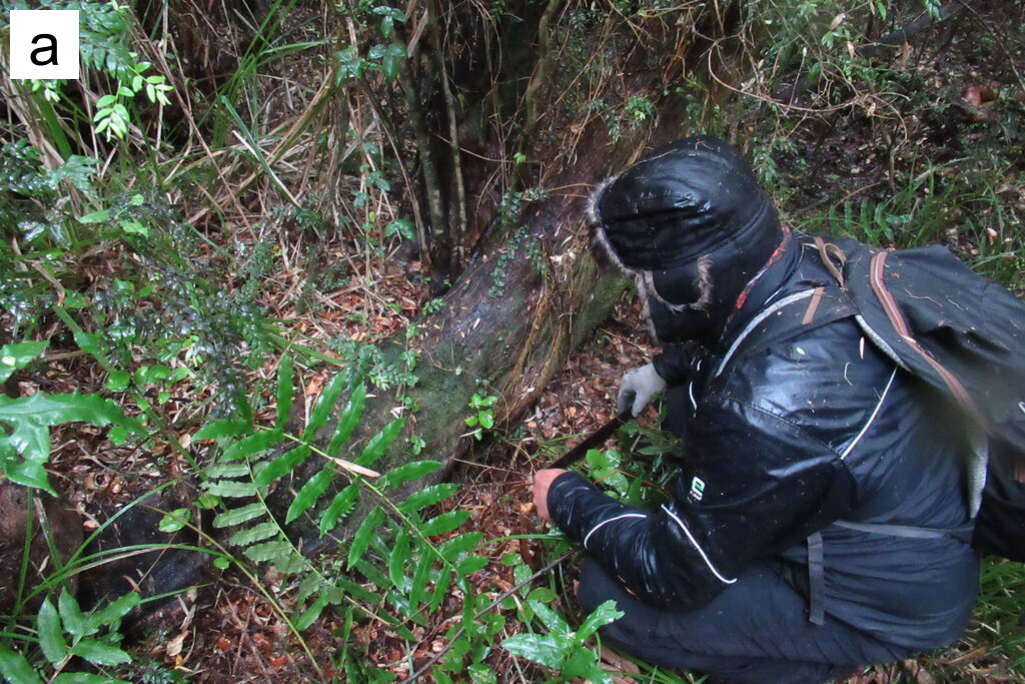
Microhabitat. The picture shows associated species such as *Parablechnumchilense* and *Luzuriagapolyphylla*.

**Figure 5b.**
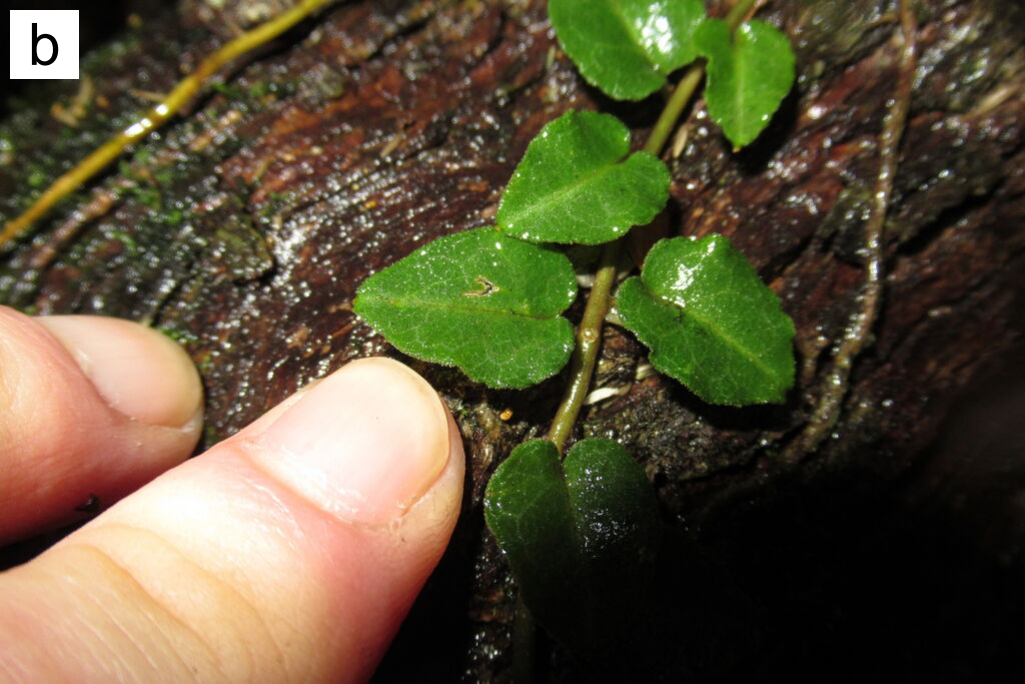
Leaves adaxial surface (photograph taken with flash)

**Figure 5c.**
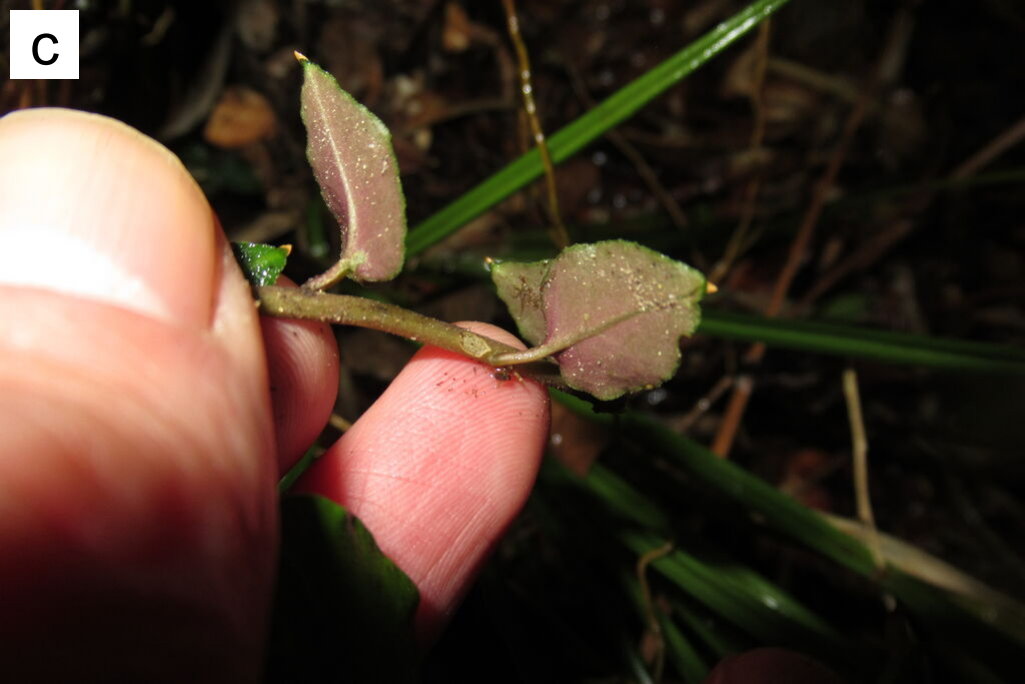
Leaves abaxial surface (photograph taken with flash)

**Figure 5d.**
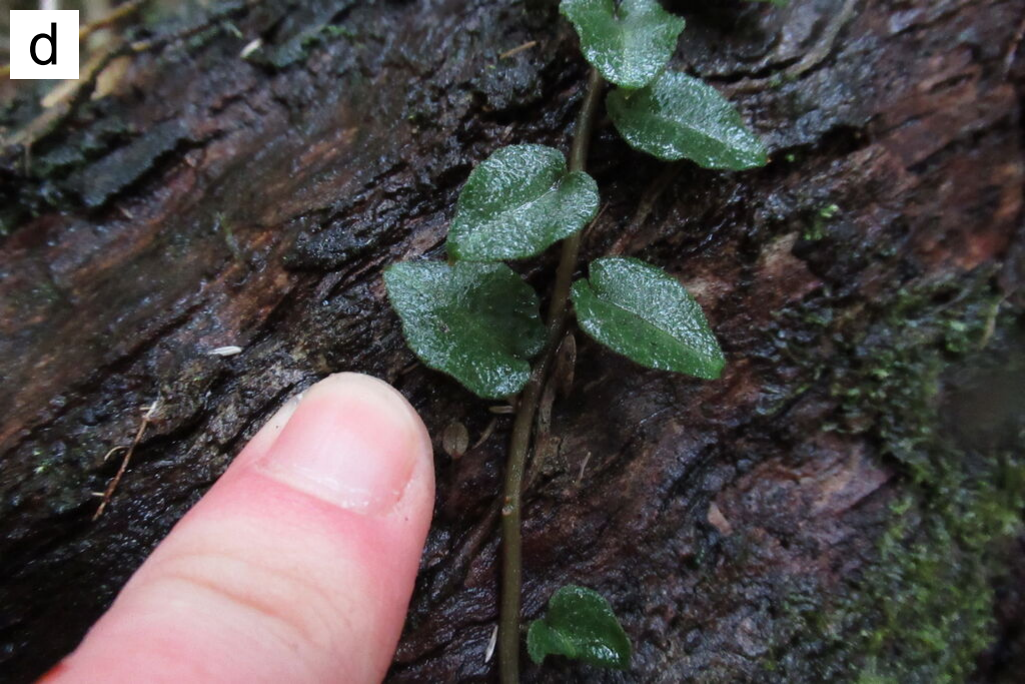
Habit in natural light
